# The Residue and Dietary Risk Assessment of Spirotetramat and Its Four Metabolites in Cabbage Using Ultra-High-Performance Liquid Chromatography–Tandem Mass Spectrometry

**DOI:** 10.3390/molecules28124763

**Published:** 2023-06-14

**Authors:** Junli Cao, Jindong Li, Pengcheng Ren, Yanli Qi, Shu Qin

**Affiliations:** Shanxi Center for Testing of Functional Agro-Products, Longcheng Campus, Shanxi Agricultural University, No. 79, Longcheng Street, Taiyuan 030031, China

**Keywords:** spirotetramat, cabbage, dissipation, dietary intake risk

## Abstract

Spirotetramat is a potential tetronic acid pesticide for controlling various pests with piercing–sucking mouthparts. To clarify its dietary risk on cabbage, we established an ultra-high-performance liquid chromatography–tandem mass spectrometry (UHPLC–MS/MS) method and then investigated the residual levels of spirotetramat and its four metabolites in cabbage collected from field experiments under good agricultural practices (GAPs). The average recoveries of spirotetramat and its metabolites in cabbage were 74~110%, while the relative standard deviation (RSD) was 1~6%, and the limit of quantitation (LOQ) was 0.01 mg kg^−1^. The terminal residue of spirotetramat was in the range of <0.05~0.33 mg kg^−1^, the chronic dietary risk (RQ_c_) was 17.56%, and the acute dietary risk (RQ_a_) was 0.025~0.049%, which means an acceptable dietary intake risk. This study provides data to guide on the use of spirotetramat and to establish the maximum residue limits (MRLs) of spirotetramat on cabbage.

## 1. Introduction

**Figure 1:**
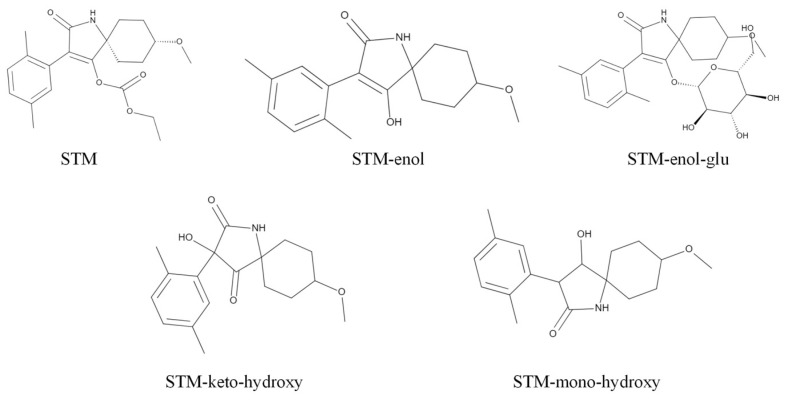
Structural formulas of spirotetramat and its metabolites.

Cabbage (*Brassica oleracea var. capitata* Linnaeus), a Brassica vegetable of the Cruciferae family, is rich in antioxidant chemicals such as vitamin C, vitamin E, flavonoids, and carotenoids, so it has the effect of reducing chronic diseases [1]. China is the largest cabbage producer in the world, with an annual yield of more than 33 million g hm^−2^ from 2000 to 2021 [2]. Spirotetramat (STM, Figure 1), *cis*-4-(ethoxycarbonyloxy)-8-methoxy-3-(2,5-xylyl)-1-azaspirodec-3-en-2-one, is a new type of tetronic acid insecticide and acaricide [3,4] which was developed by Bayer CropScience in 2008 to control aphids [5,6]. STM is the only insecticide that has the dual guiding property of moving up and down the crop through both the xylem and phloem, killing larvae by inhibiting the biosynthesis of insect fat, and can effectively control a variety of pests with piercing mouthparts and harmful mites [7]. As there is no cross-resistance to existing insecticides and little negative effect on beneficial arthropods, 140 STM products have been registered in China [8].

The European Union, Codex Alimentarius Commission (CAC), and other countries set maximum residue limits (MRLs) as thresholds for monitoring pesticide residues and ensuring food safety [9,10]. However, due to non-standardized detection methods, the existing MRLs for spirotetramat in China were all “temporary”. Establishing a sensitive method for identifying and quantifying spirotetramat in agricultural products is significant. Previous reports focused on the analysis methods of STM in fruits (apples, grapes, oranges, strawberries, mangoes), vegetables (cucumbers, Chinese cabbage, spinach, pepper, onions), and cotton by liquid chromatography or liquid chromatography–mass spectrometry [11,12,13,14,15]. However, there were some challenges in the detection of STM’s four metabolites, namely spirotetramat-enol (STM-enol), spirotetramat-enol-glucoside (STM-enol-glu), spirotetramat-keto-hydroxy (STM-keto), and spirotetramat-mono-hydroxyl (STM-mono). Han et al. determined the presence of STM and STM-enol in apple and apple processed products based on ultra-high-performance liquid chromatography–tandem mass spectrometry (UPLC–MS/MS) [16]. Mohapatra et al. and Singh et al. used the QuEChERS (Quick, Easy, Cheap, Effective, Rugged, Safe) method based on high-performance liquid chromatography (HPLC) to quantify STM and STM-enol in mango whole fruit, peel, pulp, grape, okra, brinjal, green chili, red chili, and soil [12,17,18]. However, there have been very few reports on determining the other three metabolites of STM. Only two reports demonstrated the residue and risk assessment of STM and four metabolites in citrus and pineapple [19,20]. To our knowledge, the residue and dietary risk assessment of STM and its four metabolites on cabbage have yet to be reported.

We aim to (1) establish a simple and reliable ultra-high-performance liquid chromatography–tandem mass spectrometry (UHPLC–MS/MS) method for simultaneous determination of STM and its four metabolites in cabbage, (2) study the terminal residues of STM and its metabolites in cabbage, and (3) evaluate the acute and chronic dietary risks of supervised trials median residue (STMR) in cabbage based on field data. This work will provide primary data for guiding the rational use of STM and the risk to cabbage consumers.

## 2. Results and Discussion

### 2.1. Method Validation

**Figure 2:**
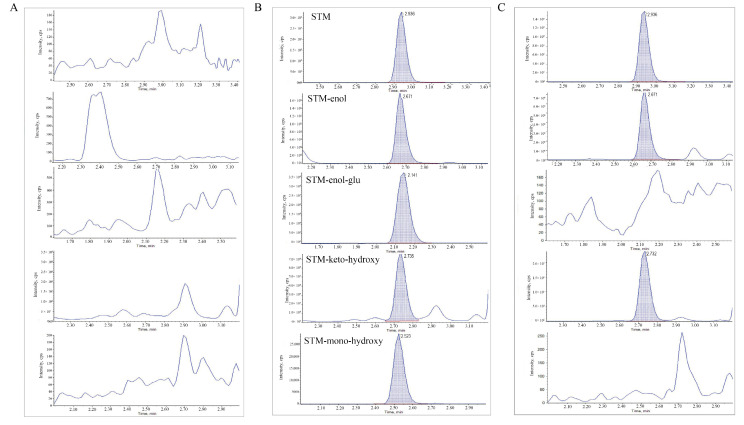
Representative UHPLC–MS/MS chromatograms: (**A**) chromatogram of blank cabbage sample, (**B**) chromatogram of spiked spirotetramat and its metabolite (0.01 mg kg^−1^) spiked in blank cabbage, and (**C**) chromatogram of field cabbage sample.

Pesticide residue analysis includes pretreatment and instrumental analysis, among which sample pretreatment in complex matrices is the most critical step. In recent years, QuEChERS pretreatment technology has been widely used to extract STM from various matrices [15,21,22,23]. However, STM-enol and STM-mono-hydroxy are more polar than STM, which may cause extraction trouble. Some improvements should be made, such as pH adjustment and using formic acid to improve recovery [24]. In this study, the modified QuEChERS method was used for the pretreatment of STM and its metabolites, using 1% formic acid acetonitrile extraction, 4 g anhydrous magnesium sulfate, 1 g sodium chloride, 1 g sodium citrate, and 0.5 g disodium hydrogen citrate for salting out, and 20 mg primary secondary amine (PSA) along with 7.5 mg graphitized carbon black (GCB) for purifying. The multiple reaction monitoring (MRM) parameters are presented in Table 1, and the chromatograms showing the separation of STM and its metabolites are shown in Figure 2.

The method of STM and its four metabolites in cabbage was verified by the linearity, correlation coefficient (R^2^), matrix effect (ME), and LOQ. The matrix-matched standard curve was constructed with the standard solution concentrations of 0.005, 0.01, 0.02, 0.05, 0.1, and 0.2 mg L^−1^ as abscissa and the corresponding chromatographic peak area as ordinate. As shown in Table 2, the determination coefficients (R^2^) of the standard curves of STM and its metabolites were greater than 0.99, indicating good linearity.

**Figure 3:**
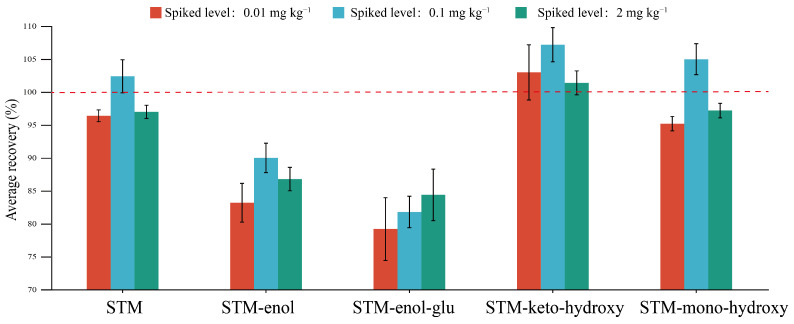
The average recovery and relative standard deviation of STM and its metabolites.

As shown in Figure 3, the average recoveries of STM were 96% to 102% at three spiked levels of 0.01, 0.1, and 2.0 mg kg^−1^, and the relative standard deviation (RSD, n = 5) was less than 2%. The average recoveries of STM-enol were between 83% and 90%, with RSD in the 2% to 3% range. The average recoveries of STM-enol-glu were between 79% and 84%, and the relative standard deviation was between 3% and 6%. The average recovery of STM-keto-hydroxy was 102~107%, and the RSD was 2~4%. The average recovery rate of STM-mono-hydroxyl was 95–105%, and the RSD was less than 2%. The average recovery of all compounds was in the range of 79% to 107%, and the RSD was less than 6%, which meets the requirements of “guideline on pesticide residue trials on crops (NY/T 788-2018)”. The LOQs of the five compounds were all 0.01 mg kg^−1^. Therefore, this method can be used for the residue analysis of STM and its metabolites in cabbage samples.

ME was caused by the co-ionization of the ESI source with other components in the matrix when analyzing the target compounds, which interferes with the quantitative accuracy of the analytes [25,26]. Except for STM (5%), the absolute ME values (Table 2) of STM-enol, STM-enol-glu, STM-keto-hydroxyl, and STM-mono-hydroxyl in cabbage were −29.2%, −49.3%, −37.1%, and −29.9%, respectively, all greater than 20%, indicating a prominent matrix weakening effect. Therefore, in this study, the matrix-matching standard curve was used for calibration as a compensation strategy for ME.

In conclusion, the modified QuEChERS pretreatment and UHPLC–MS/MS method was satisfactory for determining STM and its metabolites, so it can be used in field experiments.

### 2.2. The Terminal Residues

In 12 provinces, STM suspension was sprayed on open-field cabbage according to the recommended dosage (60 g ai hm^−2^). The terminal residues of cabbage were collected at 7 d, 10 d, and 14 d after application, and the total residue of STM was calculated. As shown in Table 3, the terminal residues of STM in cabbage were between <0.01 and 0.108 mg kg^−1^, those for STM-enol were in the range of <0.010 to 0.035 mg kg^−1^, and those for the STM-keto-hydroxyl group were less than 0.14 mg kg^−1^. The residues of STM-enol-glu and STM-mono-hydroxyl were all ≤0.01 mg kg^−1^ in actual cabbage samples. The total residue (risk assessment definition) of STM in cabbage was <0.050~0.33 mg kg^−1^, which was lower than the maximum residue limit (MRL) of STM in cabbage as stipulated by the CAC (2 mg kg^−1^) [27], European Union (7 mg kg^−1^) [28], United States (2.5 mg kg^−1^) [29], Japan (7 mg kg^−1^) [30], and Australia (7 mg kg^−1^) [31].

### 2.3. Dietary Risk Assessment

The chronic dietary risk quotient (RQ_c_) and acute dietary risk quotient (RQ_a_) were used to assess the chronic and acute dietary risk of STM intake from cabbage, respectively. According to the China Pesticide Information Network, there are 136 products registered for cabbage, celery, tomato, eggplant, chili, cucumber, potato, citrus, apple, pear, peach, banana, watermelon, melon, and tea. The national estimated daily intake (NEDI) of STM was calculated based on the dietary group diet of different populations in China published by the Ministry of Health in 2002, combined with STMR. Since the STMR of crops other than cabbage could not be obtained, the MRL of each country was chosen instead of STMR. The MRLs established by China, the Commission, the United States, and Australia should be given priority. Based on risk maximization, the maximum value is selected for evaluation when there are multiple MRL values. The average weight of the general Chinese population was 63 kg. The ADI of STM was 0.05 mg kg^−1^ bw (GB2763-2021). STMR in cabbage and MRLs in potato, celery, peach, and tea were used to calculate the NEDI of STM. As shown in Table 4, the RQ_c_ at 7, 10, and 14 days between harvesting periods was all 17.56%, much lower than 100%, indicating that the long-term dietary risk of STM would not threaten ordinary consumers.

The short-term dietary risk of STM after intake of cabbage was assessed (Table 5). According to the official data of the World Health Organization [32], the LP of cabbage in different age groups in China ranges from 0.0201 kg d^−1^ to 0.0515 kg d^−1^. The high residue of STM in cabbage was 0.33 mg kg^−1^. Therefore, in four different age groups, the national estimated short-term intake (NESTI) of STM was in the range of 2.46 × 10^−4^ to 5.39 × 10^−4^ mg (kg bw)^−1^. The RQ_a_ was from 0.025% to 0.054%, much lower than 100%, indicating that the short-term dietary intake risk caused by STM in children and adults after eating cabbage was acceptable. Our experiment was significant in determining the residual status of STM, providing a scientific basis for reducing the dietary risk assessment and the supervision of agricultural authorities, and protecting people’s consumption health.

## 3. Materials and Methods

### 3.1. Chemicals and Reagents

The certified standards, STM (purity 98.86%), STM-enol (purity 99.12%), STM-enol-glu (purity 95.7%), STM-keto-hydroxy (purity 94.24%), and STM-mono-hydroxyl (purity 99.48%) were provided by Dr. Ehrenstorfer (Augsburg, Germany). Analytical grade acetonitrile was from Tiandi Co., Ltd. (Ohio, USA). HPLC-grade acetonitrile and formic acid were purchased from Thermo Fisher Scientific Co., Ltd. (Shanghai, China). HPLC-grade methanol was purchased from Merck, Germany. HPLC-grade ammonium acetate came from Guangfu Institute of Fine Chemical Industry (Tianjin, China). Analytical grade formic acid was provided by Sinopharm Chemical Reagent Co., Ltd. (Shanghai, China). Analytical grade anhydrous magnesium sulfate, sodium chloride, disodium hydrogen citrate, and sodium citrate were purchased from Shimadzu Technology Trading Co., Ltd. (Shanghai, China). Disperse solid phase extraction purification tubes (20 mg PSA, 7.5 mg GCB, and 142.5 mg anhydrous MgSO_4_, 2 mL) were provided by Aces Scientific. The polytetrafluoroethylene filter (0.22 μm) was purchased from GL Sciences Technology Trading Co., Ltd. (Shanghai, China).

The standard solution was prepared using acetonitrile to dissolve 10.1 mg, 10.1 mg, 10.4 mg, 10.6 mg, and 10.1 mg STM, STM-enol, STM-enol-glu, STM-keto-hydroxy, and STM-mono-hydroxyl in a 10 mL volumetric flask, respectively. These reserves were then stored in a refrigerator at −18 °C. The above standard solution was diluted with acetonitrile to obtain a mixed standard solution with a concentration of 100 g mL^−1^. Then, six mixed standard solutions with a concentration of 0.005, 0.01, 0.02, 0.05, 0.1, and 0.2 µg mL^−1^ were serially diluted with acetonitrile. Matrix-matched standards were obtained by spiking an appropriate amount of standard to blank cabbage extract.

### 3.2. Field Trials and Sampling

According to “Guideline on Pesticide Residue Trials” (NY/T788-2018), STM’s terminal residue experiment in cabbage was carried out in Jinzhong city in Shanxi province (E112°, N37°), Liaoyang city in Liaoning province (E123°, N41°), Changping District in Beijing (N40°, E116°), Qingdao city in Shandong province (N36°, E120°), Xinxiang city in Henan province (N35°, E113°), Suzhou city in Anhui province (N34°, E116°), Fengxian District in Shanghai (N30°, E121°), Liuyang city in Hunan province (N27°, E113), Nanning city in Guangxi province (N22°, E108°), Guiyang city in Guizhou province (N26°, E106°), Haikou city in Hainan province (N20°, E110°), and Foshan city in Guangdong province (N22°, E112°). These field experiments sites covered almost all the residual risks of cabbage planting areas, taking into account the effects of cabbage planting methods, varieties, soil types, cultivation methods, and climate on pesticide residues. The soil properties and climatic conditions of the field plots are presented in Table S1 (Supplementary Materials). Soil pH, cation exchange capacity, and organic matter were measured in accordance with the Agricultural Industry Standard of the People’s Republic of China—NY/T1121 Part II, Part V, and Part VI. The mean temperature data and precipitation were continuously obtained by the field meteorological station during the experimental period. In the experiment, STM treatment and one control group were set up. Two replicates were set up for each treatment, and each treatment area was about 50 m^2^. We kept a buffer zone between the treatment intervals to avoid cross-contamination. About 14 days before maturity, STM was sprayed on the cabbage according to the recommended dose (60 g ai hm^−2^). The cabbage samples collected at 7 d, 10 d, and 14 d were used as terminal residual samples. After removing the wilted part, the cabbage sample was chopped with a knife, and two samples of no less than 200 g were taken by the quartering method, one for the experimental sample and one for the backup sample. All field samples were stored in a −20 °C freezer.

### 3.3. Sample Preparations

Cabbage samples were homogenized with a pulverizer. The weighed 10.0 g cabbage sample was put into a 50 mL PTFE centrifuge tube and 10 mL acetonitrile-formic acid (99:1, *v*:*v*) was added. The tube was vortexed for 10 min to extract the target compound. Then, 4 g of anhydrous magnesium sulfate, 1 g of sodium chloride, 1 g of sodium citrate, and 0.5 g of disodium hydrogen citrate were added. Then, the tubes were shaken for 5 min and centrifuged at 8000 rpm for 5 min.

Next, 1.5 mL of the supernatant was transferred to a 2 mL centrifuge tube containing 20 mg PSA, 7.5 mg GCB, and 142.5 mg anhydrous MgSO_4_.The tube was vortexed for 3 min and then centrifuged at 5000 rpm for 2 min. Finally, a 0.22 μm organic membrane was used for filtration, to be determined by UHPLC–MS/MS.

### 3.4. UHPLC–MS/MS Analysis

A UHPLC–MS/MS system (Triple Quad 4500, AB Sciex) equipped with an electrospray ionization source was used to analyze STM and its metabolites in cabbage. The chromatographic column was a Kinetex^®^ 2.6 μm EVO C18 100 μm chromatographic column (50 × 2.1 mm). The mobile phase comprised 4 mmol/L ammonium acetate aqueous solution with 0.1% formic acid (A) and methanol (B) at a flow rate of 0.3 m L/min. The gradient elution procedure was: 0~0.5 min constant, 90% A; 2.5~3.5 min, 5% A; and 3.6~5.1 min, 90% A. The column and sample room temperatures were 40 °C and 20 °C, respectively. The electrospray ionization source was scanned in positive ion mode. The ionization voltage was 5500 V, the collision gas was nitrogen, and the temperature of the heating module was 550 °C. The injection volume was 2 μL. The mass spectrometric parameters of STM and its metabolites are shown in Table 1.

### 3.5. Method Validation

The analytical methods of STM and its metabolites in cabbage samples were verified by the accuracy, precision, linearity, limit of quantitation (LOQ), and matrix effect (ME), according to SANTE/11312/2021 [33].

To evaluate the accuracy and precision of the analytical method, the standard of STM and its metabolites were spiked to blank cabbage samples at 0.01 mg kg^−1^, 0.1 mg kg^−1^, and 2.0 mg kg^−1^, with five replicates per level. The recovery (%) and relative standard deviation (RSD, %) were calculated. The method had qualified accuracy and precision when the recovery was 70~120% and the RSD was less than 20%. The limit of quantitation (LOQ) was defined as the lowest spiked level.

The linearity was evaluated by analyzing the standard and matrix-matched standard solution curves in the concentration range of 0.005–0.2 mg L^−1^. The matrix effect (ME) was calculated by comparing the slope of the matrix-matched calibration curve to the slope of the solvent calibration curve by the following formula:

ME (%) = (S_m_ − S_s_)/S_s_ × 100% (1)

where S_m_ and S_s_ represent the slopes of the matrix-matched standard curve and the solvent standard curve, respectively. A positive ME value represents a matrix-enhancing effect, while a negative ME value shows a matrix-inhibiting effect. When the matrix effect is in the range of −20~20%, the matrix effect can be ignored; when the matrix effect is in the range of −50~−20% or 20~50%, it means a weak matrix effect; and when the matrix effect is lower than -50% or greater than 50%, it represents a strong matrix effect.

### 3.6. Definition of STM Residue

The residue definition for risk assessment of STM in plant foods was proposed as the “Sum of spirotetramat, STM-enol, STM-keto-hydroxy, STM-mono-hydroxyl, and STM-enol-glu, expressed as spirotetramat”. In this study, the definition was the sum of STM and its four metabolites, which were expressed as spirotetramat. The sum of STM was calculated as follows:

C_sum_ = C_STM_ + 1.239 × C_STM-enol_ + 0.806 × C_enol-glu_ + 1.177 × C_keto-hydroxy_ + 1.231 × C_mono-hydroxy_ (2)

where CSTM, C_eno_, C_eno-glu_, C_keto-hydroxy_, and C_mono-hydroxy_ were the residue concentrations of STM, STM-enol, STM-enol-glu, STM-keto-hydroxy, and STM-mono-hydroxy, respectively. The values 1.239, 0.806, 1.177, and 1.231 represent the ratio of the molecular weight of STM-enol, STM-enol-glu, STM-keto-hydroxy, and STM-mono-hydroxy to spirotetramat, respectively. When the residual concentration was less than the limit of quantitation (LOQ), 0.01 mg kg^−1^ was used for calculation.

### 3.7. Dietary Risk Assessment

In recent reports, the risk quotient method was used to assess the chronic dietary risk (RQ_c_) and acute dietary risk (RQ_a_) of pesticides. An RQ < 100% indicates that the dietary risk is acceptable to consumers, while an RQ > 100% indicates an unacceptable risk.

RQ_c_ was the ratio of the NEDI to ADI and was calculated as follows:

NEDI = Fi × STMR/BW(3)

RQ_c_ = NEDI/ADI (4)

where NEDI is the national estimated daily intake, (mg kg^−1^ bw) d^−1^; FI is the per capita daily intake of cabbage, kg d^−1^; and STMR is the median residue of STM in cabbage obtained from field experiments, mg kg^−1^. The field experiment showed that the STMR of STM was 0.051 (PHI = 7), 0.050 (PHI = 10), and 0.050 (PHI = 14) respectively. BW is the average body weight of Chinese adult, 63 kg. ADI represents the allowable daily intake, (mg kg^−1^ bw) d^−1^. The ADI of STM is considered to be 0.05 mg kg^−1^ bw [34,35].

RQ_a_ was calculated as a percentage of NESTI to ARfD:

NESTI = Lp × HR/bw(5)

RQ_a_ = NESTI/ARfD (6)

where NESTI is the national estimated short-term intake (mg kg^−1^ bw); LP is the highest consumption of cabbage per day, kg d^−1^; HR is the highest terminal residue (0.33 mg kg^−1^) of STM in cabbage obtained from field trials; BW is the average body weight of different age groups; and ARfD is the acute reference dose. The ARfD of STM was 1 mg kg^−1^ bw d^−1^ [35].

## 4. Conclusions

We verified a simple, sensitive, reliable quantitative method for determining STM and its four metabolites. The samples were extracted with acetonitrile-formic acid, purified by PSA and GCB, and determined qualitatively and quantitatively by UHPLC–MS/MS. The method’s precision, accuracy, linearity, and LOQ all meet the requirements of the guidelines for pesticide residue analysis. In the supervised field experiment, the terminal residue range of STM was from <0.05 mg kg^−1^ to 0.033 mg kg^−1^. The chronic dietary risk was 17.56%, and the acute dietary risk was 0.025~0.049%, all of which are less than 100%, indicating that the STM suspending agent is acceptable to the chronic dietary risk of cabbage and was sprayed according to the active ingredient at 60 g hm^−2^.

## Figures and Tables

**Table 1:** MRM conditions of UHPLC–MS/MS for spirotetramat and its metabolites.

Compound	Retention Time (R_t_, min)	Production (m z^−1^)	Declustering Potential (DP, V)	Collision Energy (CE, V)
STM	2.91	374.20 > 330.1 (quantitation)	66	47
		374.20 > 216.1 (confirmation)		21
STM-enol	2.65	302.30 > 270.2 (confirmation)	60	40
		302.30 > 216.0 (quantitation)		30
STM-enol-glu	2.10	464.40 > 302.2 (confirmation)	67	20
		464.40 > 216.0 (quantitation)		40
STM-keto-hydroxy	2.71	318.20 > 214.0 (quantitation)	40	20
		318.20 > 268.1 (confirmation)		20
STM-mono-hydroxy	2.49	304.30 > 254.1 (confirmation)	60	20
		304.30 > 211.1 (quantitation)		20

**Table 2:** The calibration curves, determination coefficient (R^2^), and matrix effect of STM and its metabolites.

Compounds	Matrix	Calibration Curve	R^2^	Matrix Effect (%)
STM	Acetonitrile	y = 4.089 × 10^6^x + 355.0	0.9998	-
	Cabbage	y = 4.292 × 10^6^x + 389.9	0.9998	5.0
STM-enol	Acetonitrile	y = 9.598 × 10^7^x + 1.015 × 10^5^	0.9979	-
	Cabbage	y = 4.865 × 10^7^x − 4125	0.9979	−29.2
STM-enol-glu	Acetonitrile	y = 1.627 × 10^7^x + 3.406 × 10^4^	0.9916	-
	Cabbage	y = 1.023 × 10^7^x + −813.7	0.9909	−49.3
STM-keto-hydroxy	Acetonitrile	y = 2.822 × 10^7^x + 2.228 × 10^4^	0.9989	-
	Cabbage	y = 1.962 × 10^7^x + 3.421 × 10^4^	0.9994	−37.1
STM-mono-hydroxy	Acetonitrile	y = 1.322 × 10^7^x + 2.637 × 10^4^	0.9953	-
	Cabbage	y = 9.614 × 10^6^x + 1474	0.9981	−29.9

**Table 3:** Terminal residues of STM in cabbage.

Locations	Pre-Harvest Interval (Days)	Mean Residues (mg kg^−1^)					
		STM	STM-enol	STM-enol-glu	STM-keto-hydroxy	STM-mono-hydroxy	Total Residues
Shanxi	7	0.039, 0.108	0.019, 0.035	<0.010, <0.010	0.081, 0.14	<0.010, <0.010	0.18, 0.33
	10	0.043, 0.073	<0.010, <0.010	<0.010, <0.010	0.13, 0.11	<0.010, <0.010	0.22, 0.24
	14	0.023, 0.077	<0.010, <0.010	<0.010, <0.010	0.049, 0.051	<0.010, <0.010	0.11, 0.17
Liaoning	7	<0.010, <0.010	<0.010, <0.010	<0.010, <0.010	<0.010, <0.010	<0.010, <0.010	<0.050, <0.050
	10	<0.010, <0.010	<0.010, <0.010	<0.010, <0.010	<0.010, <0.010	<0.010, <0.010	<0.050, <0.050
	14	<0.010, <0.010	<0.010, <0.010	<0.010, <0.010	<0.010, <0.010	<0.010, <0.010	<0.050, <0.050
Beijing	7	<0.010, <0.010	<0.010, <0.010	<0.010, <0.010	0.011, <0.010	<0.010, <0.010	0.052, <0.050
	10	<0.010, <0.010	<0.010, <0.010	<0.010, <0.010	<0.010, <0.010	<0.010, <0.010	<0.050, <0.050
	14	<0.010, <0.010	<0.010, <0.010	<0.010, <0.010	<0.010, <0.010	<0.010, <0.010	<0.050, <0.050
Shandong	7	<0.010, <0.010	<0.010, <0.010	<0.010, <0.010	<0.010, <0.010	<0.010, <0.010	<0.050, <0.050
	10	<0.010, <0.010	<0.010, <0.010	<0.010, <0.010	<0.010, <0.010	<0.010, <0.010	<0.050, <0.050
	14	<0.010, <0.010	<0.010, <0.010	<0.010, <0.010	<0.010, <0.010	<0.010, <0.010	<0.050, <0.050
Henan	7	<0.010, <0.010	<0.010, <0.010	<0.010, <0.010	<0.010, <0.010	<0.010, <0.010	<0.050, <0.050
	10	<0.010, <0.010	<0.010, <0.010	<0.010, <0.010	<0.010, <0.010	<0.010, <0.010	<0.050, <0.050
	14	<0.010, <0.010	<0.010, <0.010	<0.010, <0.010	<0.010, <0.010	<0.010, <0.010	<0.050, <0.050
Anhui	7	<0.010, <0.010	<0.010, <0.010	<0.010, <0.010	0.033, 0.032	<0.010, <0.010	0.078, 0.078
	10	<0.010, <0.010	0.01, 0.02	<0.010, <0.010	0.032, 0.028	<0.010, <0.010	0.08, 0.087
	14	<0.010, <0.010	<0.010, <0.010	<0.010, <0.010	0.012, 0.014	<0.010, <0.010	0.055, 0.057
Shanghai	7	<0.010, <0.010	<0.010, <0.010	<0.010, <0.010	<0.010, <0.010	<0.010, <0.010	<0.050, <0.050
	10	<0.010, <0.010	<0.010, <0.010	<0.010, <0.010	<0.010, <0.010	<0.010, <0.010	<0.050, <0.050
	14	<0.010, <0.010	<0.010, <0.010	<0.010, <0.010	<0.010, <0.010	<0.010, <0.010	<0.050, <0.050
Hunan	7	0.018, <0.010	<0.010, <0.010	<0.010, <0.010	<0.010, <0.010	<0.010, <0.010	0.058, <0.050
	10	0.021, 0.039	<0.010, <0.010	<0.010, <0.010	<0.010, <0.010	<0.010, <0.010	0.061, 0.079
	14	<0.010, <0.010	<0.010, <0.010	<0.010, <0.010	<0.010, <0.010	<0.010, <0.010	<0.050, <0.050
Guangxi	7	<0.010, <0.010	<0.010, <0.010	<0.010, <0.010	0.012, 0.016	<0.010, <0.010	0.054, 0.058
	10	<0.010, <0.010	<0.010, <0.010	<0.010, <0.010	<0.010, <0.010	<0.010, <0.010	<0.050, <0.050
	14	<0.010, <0.010	<0.010, <0.010	<0.010, <0.010	<0.010, <0.010	<0.010, <0.010	<0.050, <0.050
Guizhou	7	<0.010, <0.010	<0.010, <0.010	<0.010, <0.010	<0.010, <0.010	<0.010, <0.010	<0.050, <0.050
	10	<0.010, <0.010	<0.010, <0.010	<0.010, <0.010	<0.010, <0.010	<0.010, <0.010	<0.050, 0.053
	14	<0.010, <0.010	<0.010, <0.010	<0.010, <0.010	<0.010, <0.010	<0.010, <0.010	<0.050, <0.050
Hainan	7	0.010, 0.013	<0.010, <0.010	<0.010, <0.010	0.028, 0.023	<0.010, <0.010	0.073, 0.069
	10	0.008, 0.012	<0.010, <0.010	<0.010, <0.010	0.028, 0.03	<0.010, <0.010	0.073, 0.077
	14	<0.010, <0.010	<0.010, <0.010	<0.010, <0.010	<0.010, <0.010	<0.010, <0.010	0.053, 0.065
Guangdong	7	<0.010, <0.010	<0.010, <0.010	<0.010, <0.010	0.01, 0.014	<0.010, <0.010	0.052, 0.056
	10	<0.010, <0.010	<0.010, <0.010	<0.010, <0.010	<0.010, <0.010	<0.010, <0.010	<0.050, <0.050
	14	<0.010, <0.010	<0.010, <0.010	<0.010, <0.010	<0.010, <0.010	<0.010, <0.010	<0.050, <0.050

**Table 4:** Chronic dietary intake risk assessment of STM based on Chinese dietary composition.

Crops	Food Classification	Fi (kg)	Residue (mg kg^−1^)	Sources	NEDI (mg)	ADI (mg)	Risk Quotient (RQ_c_, %)
Potato	Tubers	0.0495	0.8	China, MRL	3.960 × 10^−2^	0.05	1.26
Celery	Dark vegetables	0.0915	4	China, MRL	3.660 × 10^−1^		11.62
Peach	Fruits	0.0457	3	China, MRL	1.371 × 10^−1^		4.35
Tea	Salt	0.012	0.1	Australia, MRL	1.200 × 10^−3^		0.04
Cabbage	Light vegetables	0.1837	0.051	STMR1 (PHI = 7)	9.369 × 10^−3^		0.30
			0.050	STMR2 (PHI = 14)	9.185 × 10^−3^		0.29
			0.050	STMR3 (PHI = 21)	9.185 × 10^−3^		0.29
	Total	0.3824			5.533 × 10^−1^ (PHI = 7)		17.56 (PHI = 7)
					5.531 × 10^−1^ (PHI = 14)		17.56 (PHI = 14)
					5.531 × 10^−1^ (PHI = 21)		17.56 (PHI = 21)

**Table 5:** Acute dietary risk assessment of STM on cabbage for 4 representative ages.

Age	Weight (kg)	Food Consumption (kg d^−1^)	NESTI (mg (kg bw)^−1^)	RQ_a_ (%)
2~10	12.3~22.9	0.0201~0.0343	4.94 × 10^−4^~5.39 × 10^−4^	0.049~0.054
11~17	34.0~46.9	0.0381~0.0440	3.10 × 10^−4^~3.70 × 10^−4^	0.031~0.037
18~59	52.1~64.9	0.0448~0.0515	2.62 × 10^−4^~2.84 × 10^−4^	0.026~0.028
≥60	51.0~61.5	0.0380~0.0472	2.53 × 10^−4^~2.46 × 10^−4^	0.025~0.025

## Data Availability

The data presented in this study are available from the authors upon request.

## Supplementary Materials

The following supporting information can be downloaded at: https://www.mdpi.com/article/10.3390/molecules28124763/s1, Table S1: Soil properties and climate conditions for field trials; Table S2: Terminal residues of STM in cabbage on 3d.

## Sample Availability

Samples from our study are available from the authors upon request.

## References

[1] Franzke A., Lysak M.A., Al-Shehbaz I.A., Koch M.A., Mummenhoff K. (2011). Cabbage family affairs: The evolutionary history of Brassicaceae. Trends Plant Sci..

[2] Food and Agriculture Organization of the United Nations https://www.fao.org/faostat/en/#compare.

[3] European Food Safety Authority (2013). Conclusion on the peer review of the pesticide risk assessment of the active substance spirotetramat. EFSA J..

[4] Vang L.E., Opperman C.H., Schwarz M.R., Davis E.L. (2016). Spirotetramat causes an arrest of nematode juvenile development. Nematology.

[5] Nauen R., Reckmann U., Thomzik J., Thielert W. (2008). Biological profile of spirotetramat (Movento^®^)—A new two-way systemic (ambimobile) insecticide against sucking pest species. Bayer Crop. J..

[6] Pesticides A., Authority V.M. (2009). Evaluation of the New Active SPIROTETRAMAT in the Product MOVENTO 240 SC INSECTICIDE.

[7] Salazar-López N.-J., Aldana-Madrid M.-L., Silveira-Gramont M.-I., Aguiar J.-L. (2016). Spirotetramat—An alternative for the control of parasitic sucking insects and its fate in the environment. Insecticide Resistance.

[8] China Pesticide Information Network. http://www.chinapesticide.org.cn/zwb/dataCenter.

[9] Ambrus A., Yang Y.Z. (2016). Global harmonization of maximum residue limits for pesticides. J. Agric. Food Chem..

[10] Winter C.K., Jara E.A. (2015). Pesticide food safety standards as companions to tolerances and maximum residue limits. J. Integr. Agric..

[11] Cui S., Li Z., Cheng G., Li R., Wang Y., Zhang X., Zhao F. (2018). Determination of sulfoxaflor, pyrifluquinazon and spirotetramat residues in fruits and vegetables by UPLC-MS/MS. Shipin Kexue/Food Sci..

[12] Mohapatra S., Deepa M., Lekha S., Nethravathi B., Radhika B., Gourishanker S. (2012). Residue dynamics of spirotetramat and imidacloprid in/on mango and soil. Bull. Environ. Contam. Toxicol..

[13] Pandiselvi S., Sathiyanarayanan S., Ramesh A. (2010). Determination of spirotetramat and imidacloprid residues in cotton seed, lint, oil and soil by HPLC UV method and their dissipation in cotton plant. Pestic. Res. J..

[14] Kumar B., Kumaran N., Kuttalam S. (2009). Determination of harvest time residues of spirotetramat on cotton using HPLC. J. Plant Prot. Environ..

[15] Li S., Liu X., Dong F., Xu J., Xu H., Hu M., Zheng Y. (2016). Chemometric-assisted QuEChERS extraction method for the residual analysis of thiacloprid, spirotetramat and spirotetramat’s four metabolites in pepper: Application of their dissipation patterns. Food Chem..

[16] Han Y., Xu J., Dong F., Li W., Liu X., Li Y., Kong Z., Zhu Y., Liu N., Zheng Y. (2013). The fate of spirotetramat and its metabolite spirotetramat-enol in apple samples during apple cider processing. Food Control.

[17] Mohapatra S., Kumar S., Prakash G. (2015). Residue evaluation of imidacloprid, spirotetramat, and spirotetramat-enol in/on grapes (Vitis vinifera L.) and soil. Environ. Monit. Assess..

[18] Singh B., Mandal K., Sahoo S.K., Bhardwaj U., Battu R.S. (2013). Development and validation of an HPLC method for determination of spirotetramat and spirotetramat cis enol in various vegetables and soil. J. AOAC Int..

[19] Zhang Q., Chen Y., Wang S., Yu Y., Lu P., Hu D., Yang Z. (2018). Dissipation, residues and risk assessment of spirotetramat and its four metabolites in citrus and soil under field conditions by LC-MS/MS. Biomed. Chromatogr..

[20] Liang Y., Wu W., Cheng X., Hu J. (2020). Residues, fate and risk assessment of spirotetramat and its four metabolites in pineapple under field conditions. Int. J. Environ. Anal. Chem..

[21] Faraji M., Noorbakhsh R., Shafieyan H., Ramezani M. (2018). Determination of acetamiprid, imidacloprid, and spirotetramat and their relevant metabolites in pistachio using modified QuEChERS combined with liquid chromatography-tandem mass spectrometry. Food Chem..

[22] Lan F., Yao J., Zhou X., Liu C., Li X., Lu Z., Jiang W., Wang Z. (2019). Residue of spirotetramat, diafenthiuron and their metabolites in fruits and vegetables by QuEChERS-ultra performance liquid chromatography-tandem mass spectrometry. Chin. J. Pestic. Sci..

[23] Zhu Y., Liu X., Xu J., Dong F., Liang X., Li M., Duan L., Zheng Y. (2013). Simultaneous determination of spirotetramat and its four metabolites in fruits and vegetables using a modified quick, easy, cheap, effective, rugged, and safe method and liquid chromatography/tandem mass spectrometry. J. Chromatogr. A.

[24] Food and Agriculture Organization (2013). Spirotetramat. https://www.fao.org/fileadmin/templates/agphome/documents/Pests_Pesticides/JMPR/Report13/5.32_SPIROTETRAMAT__234_.pdf.

[25] Liu H.C., Lin D.L., McCurdy H.H. (2013). Matrix Effects in the Liquid Chromatography-Tandem Mass Spectrometry Method of Analysis. Forensic Sci. Rev..

[26] Hayama T. (2020). Matrix Effects in Mass Spectrometry Analysis. Anal. Sci..

[27] Codex Online Databases. https://www.fao.org/fao-who-codexalimentarius/codex-texts/dbs/pestres/pesticide-detail/en/?p_id=234.

[28] EU Pesticide Database. https://ec.europa.eu/food/plant/pesticides/eu-pesticides-database/start/screen/mrls.

[29] United States Pesticide MRLs. https://bcglobal.bryantchristie.com/db#/pesticides/results?q=eyJmaWx0ZXJzIjp7ImZVU1NlY3Rpb24xOFJlZ2lvbmFsIjowLCJmRmFjaWxpdHlVc2UiOjIsImZJbXBvcnRUb2xlcmFuY2UiOjAsInR5cGVTb3J0T3JkZXIiOlsxNSwyLDFdfSwiYm9keSI6W3siaXRlbVR5cGVJRCI6MTUsImxpc3QiOls0MzY2XX0seyJpdGVtVHlwZUlEIjoyLCJsaXN0IjpbNDU4XX0seyJpdGVtVHlwZUlEIjoxLCJsaXN0IjpbMjFdfV19&isSimpleViewProp=true.

[30] The Japen Food Chemical Research Foundation http://db.ffcr.or.jp/front/.

[31] Australian Goverment https://www.legislation.gov.au/Details/F2023C00438.

[32] World Health Organization Template for the Evaluation of Acute Exposure (IESTI). https://cdn.who.int/media/docs/default-source/food-safety/gems-food/guidance-iesti-2014.pdf?sfvrsn=9b24629a_2.

[33] European Commission (2021). Guidance Document on Analytical Qualitycontrol and Method Validation Procedures for Pesticides Residues Andanalysis in Food and Feed. https://www.eurl-pesticides.eu/userfiles/file/EurlALL/SANTE_11312_2021.pdf.

[34] (2021). National Food Safety Standard-Maximum Residue Limits for Pesticides in Food.

[35] Anastassiadou M., Bernasconi G., Brancato A., Cabrera L.C., Greco L., Jarrah S., Kazocina A., Leuschner R., Magrans J.O., Miron I. (2020). Review of the existing maximum residue levels for spirotetramat according to Article 12 of Regulation (EC) No 396/2005. EFSA J..

