# Supplementary material for: The Residue and Dietary Risk Assessment of Spirotetramat and Its Four Metabolites in Cabbage Using Ultra-High-Performance Liquid Chromatography–Tandem Mass Spectrometry
Source: Molecules. 2023 Jun 14;28(12):4763. doi: 10.3390/molecules28124763 (PMC10302016; doi:10.3390/molecules28124763)
Supplement: Supplementary file 1 [file molecules-28-04763-s001.zip › molecules-2413768-supplementary.pdf]

Table S1. Soil properties and climate conditions for field trials.

Site	soil properties			Average temperature (°C)	Total precipitation (mm)
	pH	Cation exchange capacity (cmol kg^{-1})	Organic content (%)		
Shanxi	8.32	10.28	1.58	21.5	39.0
Liaoning	6.90	19.00	3.10	23.4	0.0
Beijing	7.47	21.10	1.51	25.8	65.8
Shandong	5.79	17.20	1.21	20.6	37.9
Henan	7.90	7.68	1.29	24.0	0.0
Anhui	7.40	14.20	1.14	19.5	106.5
Shanghai	8.43	34.51	2.49	26.1	63.2
Hunan	5.00	3.10	1.63	28.2	1.6
Guangxi	6.70	5.60	3.18	17.9	10.0
Guizhou	6.51	24.80	4.30	17.2	54.3
Hainan	5.40	4.13	1.41	19.0	0.0
Guangdong	5.90	3.90	1.02	25.2	70.5

Table S2. Terminal residues of STM in cabbage on 3d.

Locations	Mean resiudes (mg/kg)											
	STM		STM-enol		STM-enol-glu		STM-keto		STM-mono		Total	
	1	2	1	2	1	2	1	2	1	2	1	2
Shanxi	0.36	0.16	0.062	0.052	<0.010	<0.010	0.23	0.15	<0.010	<0.010	0.73	0.41
Beijing	0.029	0.011	0.010	<0.010	<0.010	<0.010	0.027	0.018	<0.010	<0.010	0.093	0.062
Anhui	0.077	0.114	<0.010	<0.010	<0.010	<0.010	0.095	0.104	<0.010	<0.010	0.22	0.27
Guangxi	<0.010	<0.010	<0.010	<0.010	<0.010	<0.010	0.010	<0.010	<0.010	<0.010	0.052	<0.050
